# The Role of Long Non-Coding RNAs in Osteosarcoma

**DOI:** 10.3390/ncrna4010007

**Published:** 2018-03-08

**Authors:** Maria Anna Smolle, Martin Pichler

**Affiliations:** 1 Department of Orthopaedics and Trauma, Medical University of Graz, Auenbruggerplatz 5, 8036 Graz, Austria; maria.smolle@cbmed.at; 2 Division of Clinical Oncology, Internal Medicine, Medical University of Graz, Auenbruggerplatz 15, 8036 Graz, Austria; 3 Division of Cancer Medicine, MD Anderson Cancer Center, 1515 Holcombe Blvd., Houston, TX 77030, USA

**Keywords:** lncRNA, osteosarcoma, pathogenesis

## Abstract

Long non-coding RNAs (lncRNAs) constitute non-protein coding transcripts with a size > than 200 nucleotides. They are involved in many cellular processes, such as chromatin remodelling, transcription, and gene expression. They play a role in the development, progression, and invasion of many human cancers, including osteosarcoma. This rare tumor entity predominantly arises in children and young adults. Treatment consists of polychemotherapy and surgical resection, increasing survival rates up to 60%. In the present review, the role of lncRNAs with prognostic, predictive, therapeutic, and diagnostic significance in osteosarcoma is discussed. Moreover, their potential application in clinical practice is highlighted.

## 1. Introduction

Osteosarcoma is a rare tumor entity especially affecting children and young adults [1]. Treatment consists of neoadjuvant and adjuvant polychemotherapy and wide en bloc resection of the tumor in between the chemotherapy cycles. In comparison to a surgical approach only, a multimodal treatment approach raises the disease-free survival probability from 10–20% up to 60% [1]. The chemotherapeutic protocol comprises the antiproliferative drugs doxorubicin, cisplatin, methothrexate in combination with folinic acid (leucovorin) and the antiproliferative drug ifosfamide, albeit the optimal combination has not been identified yet [1]. 

Non-coding RNAs are a class of non-protein coding transcripts which can be divided by their function or size [2]. Short non-coding RNAs include microRNAs, transferRNAs, piwiRNAs, and others. MicroRNAs have been involved in many types of cancer and are master regulators of gene expression and cancer development [3,4]. On the other hand, long non-coding RNAs (lncRNAs) constitute non-protein coding RNA molecules with a size of more than 200 nucleotides [5,6]. They are involved in various processes, including gene expression, chromatin remodelling, post-transcriptional processing, and transcription [7,8]. Long non-coding RNAs are frequently aberrantly expressed in human cancers and they promote tumor development, progression, and metastasis [9,10,11,12,13,14]. Therefore, they can serve as predictive, prognostic, diagnostic, and therapeutic biomarkers [15,16,17]. With regards to their clinical application, lncRNAs can be targeted and deleted/amplified via different ways; their expression can be enhanced by using lentiviral vector construction transfected with the cDNA of the lncRNA of interest [18]. On the other hand, expression of lncRNAs can be reduced via degradation of the RNA by RNA interference (RNAi), application of CRIPR/Cas9 genome editing or degradation of RNA by RNase H activate antisense oligonucleotides (ASOs) [19]. RNAi uses a multiprotein RNAi-induced silencing complex (RISC) containing a small interfering RNA (siRNA) [19]. With CRISPR-Cas9 genome editing, specific CRISPR RNAs (crRNAs) hybridized to the trans-activating crRNA (tracrRNA) are complexed to the Cas9 protein. With this method, likewise small (lncRNA-21A) and large (UCA1) lncRNAs can be removed from the genome [19,20]. As a third option, the RNase H-mediated antisense RNA knockdown capitalising on the engodenous RNase H1 enzyme can be used to knock down lncRNAs [19,21].

The following review will provide an overview of lncRNAs involved in the pathogenesis, progression, and potential treatment of human osteosarcoma (Table 1). 

## 2. Long Non-Coding RNAs in Osteosarcoma; Real-Time Quantitative Polymerase Chain Reaction

### 2.1. Prognostic Biomarkers

The lncRNA *Maternally expressed gene 3* (*MEG3*) is under-expressed in several human cancers, including non-small cell lung cancer, colorectal cancer and osteosarcoma [22,36,37]. It is regulated by lncRNA *Ewing-sarcoma associated transcript-1* (*EWSAT1*) and downregulation of *MEG3* in the presence of *EWSAT1* leads to proliferation, invasion, and cellular migration of osteosarcoma cells, based on gain- and loss-of-function assays [38]. Consequently, decreased expression of *MEG3* in human osteosarcoma tissue is associated with advanced clinical stage (I/II vs. III) and presence of distant metastasis. Moreover, low *MEG3*-levels correlate with poor overall survival (OS) in osteosarcoma patients [22]. Therefore, *MEG3* could serve as a prognostic tissue biomarker in osteosarcoma, with high levels indicating a good prognosis.

Other than MEG3, lncRNA *Highly upregulated in liver cancer* (*HULC*) is upregulated in human osteosarcoma [39]. High levels of *HULC* are associated with advanced clinical stage (IIA vs. IIB/III), presence of distant metastasis and poor in OS. In osteosarcoma cell lines MG-63, U2OS, OS-732, and Saos-2, *HULC* is significantly overexpressed as compared with the primary osteoblast cell-line hFOB1.19 [23,39]. Knockdown of *HULC* via transfection of short-hairpin RNA targeted HULC #2 (sh-HULC#2, GenePharma, Shanghai, China) reduces cellular proliferation, migration and invasion, and induces apoptosis by acting as a sponge for microRNA-122 (miR-122) in MG63 and OS-732 cell lines [23]. On the other hand, the upregulation of miR-122 via transfection of OS-732 cell line with miR-122 mimics results in inactivation of JAK/STAT, NOTCH, and PI3K/AKT-pathways. Consequently, *HULC* may not only serve as a prognostic tissue biomarker in human osteosarcoma as high HULC-levels are associated with advanced clinical stage and presence of metastasis, but also constitutes a potential therapeutic target, since experimental knockdown reduces the migratory potential of osteosarcoma cell lines.

The lncRNA *Taurine upregulated gene 1* (*TUG1*) is likewise upregulated in human osteosarcoma tissue as compared to adjacent normal tissue [34]. *Taurine upregulated gene 1*-overexpression measured via quantitative polymerase chain reaction (qPCR) is significantly associated with a tumor size exceeding 8 cm, advanced clinical stage and administration of adjuvant chemotherapy. Moreover, high levels of *TUG1* significantly correlate with poor progression-free and overall-survival. Interestingly, *TUG1* was found at significantly lower levels in serum samples of osteosarcoma-patients following surgery in comparison to serum samples taken preoperatively. Moreover, *TUG1* plasma-levels correlate with disease status, with elevated levels indicating disease progression or recurrence. Of note, knockdown of *TUG1* via using small-interfering RNA targeting *TUG1* results in decreased cell proliferation, colony formation, enhanced apoptosis, and induced cell-cycle arrest in the G1/S-phase in U2OS and Saos-2 cell lines [33]. In the same cell lines, *TUG1* acts as an endogenous sponge to directly bind miR-9-5p and thus reduce its expression. Moreover, it diminishes expression of POU class 2 homeobox 1 (POU2F1), thus acting in a TUG1/mir-p-5p/POU2F1-axis in osteosarcoma cells (Saos-2 and U2OS cell lines) [33]. Taken together, *TUG1* may not only serve as a prognostic and therapeutic, but also diagnostic and surveillance-marker in patients with osteosarcoma.

As with HULC and TUG1, lncRNA *Activated by transforming growth factor-beta* (*lncRNA-ATB*) is involved in osteosarcoma pathogenesis via regulation of a miRNA [24]. Serum levels of *lncRNA-ATB* are not only increased in patients with osteosarcoma, but are also overexpressed in osteosarcoma tissues and cell lines U2OS, SAOS2, MG-63, and HOS. Moreover, *lncRNA-ATB* targets miR-200s, thus upregulating miR-200s target genes *Zinc finger E-box-binding homeobox 1 and 2* (*ZEB1/2*) [24]. Overexpression of *lncRNA-ATB* results in enhanced cell proliferation, migration and invasion in the MG63 cell line. Clinically, high levels of *LncRNA-ATB* correlate with presence of metastasis, advanced tumor stage and local recurrence [24]. Therefore, *lncRNA-ATB* may be used both as a prognostic and diagnostic biomarker in the management of osteosarcoma.

Another lncRNA targeting ZEB1 and miR-200s is *ZEB1 antisense 1* (*ZEB1-AS1*), which is overexpressed in human osteosarcoma tissues [31]. High *ZEB1-AS1*-levels are associated with advanced tumor stage, a tumor size > 8 cm, presence of metastasis, poor recurrence-free survival, and poor OS. Experimentally, overexpression of *ZEB1-AS1* in the osteosarcoma cell line HOS via transfection with pcDNA3.1-ZEB1-AS1 plasmid results in enhanced cellular proliferation and migration, whilst knockdown of *ZEB1-AS1* by transfection with short-hairpin (shRNA)-ZEB1-AS1 has the opposite effect. *ZEB1-AS1* is the antisense transcript along the *ZEB1* locus and leads to expression of ZEB1 mRNA and protein in the HOS cell line. On the other hand, the knockdown of *ZEB1-AS1* results in diminished ZEB1-expression in the Saos-2 cell line [31]. Of note, ZEB1 is required to exert the proliferative and migratory role of *ZEB1-AS1*. Additionally, miR-200s-levels negatively correlate with *ZEB1-AS1* and ZEB, as *ZEB1-AS1* relieves the inhibition of ZEB1 exerted by miR-200s [40]. In clinical practice, *ZEB1-AS1* could therefore serve as a marker to indicate patients’ prognosis.

Another lncRNA upregulated in human osteosarcoma tissue is *FGFR3 antisense transcript 1* (*FGFR3-AS1*) [26]. On the cellular level, presence of *FGFR3-AS1* leads to upregulation of Fibroblast growth factor receptor 3 (FGFR3) expression via antisense pairing with the 3′ untranslated region (3′UTR) of FGFR3. Moreover, the knockdown of *FGFR3-AS1* via transfection of MG63 cell line with the FGFR3-AS1 shRNA expression plasmid pGPU6/GFP/Neo inhibits cell cycle progression and proliferation in-vitro. Moreover, tumor growth is likewise reduced in-vivo, using xenograft models implanted with FGFR3-AS1 stably knocked down MG63 cells [26]. Thus, *FGFR3-AS1* may be used in clinical practice as a molecular tissue marker for predicting prognosis of patients with osteosarcoma.

### 2.2. Predictive Biomarkers

The lncRNA *Lung cancer associated transcript 1* (*LUCAT1*) is specifically overexpressed in osteosarcoma cell-lines resistant to methotrexate (MTX), one of the most effective drugs used in treatment of patients with osteosarcoma [30]. Cell line MG63 was made MTX-resistant in a stepwise manner by exposing it to increasing doses of the chemotherapeutic drug and culturing the survived MG63/MTX cells in the conditioned medium with 1 mg/mL MTX. Additionally, the drug-resistance related protein ATP binding cassette subfamiliy B member 1 (ABCB1) is likewise overexpressed in MTX-resistant osteosarcoma cell-lines MG63/MTX and HOS/MTX. The knockdown of *LUCAT1* does not only diminish expression of resistance-related genes *MDR1, MRP5*, and *LRP1* during treatment with MTX, but also reduces the number of invasive cells. The interaction between *LUCAT1* and ABCB1 is mediated via *miR-200c*, which binds to the 3′UTR of *ABCB1* and is itself regulated by *LUCAT1* [30]. Consequently, high *LUCAT1-*levels could serve as predictive biomarkers in patients with osteosarcoma regarding MTX-sensitivity, provided that further experiments show that *LUCAT1* is also overexpressed in human osteosarcoma tissue samples. 

Long non-coding RNA *Forkhead box protein C2 antisense 1* (*FOXC2-AS1*) is overexpressed both in human osteosarcoma tissues and cell-lines MG63 and KH-OS resistant to doxorubicin (DXR), another important chemotherapeutic agent used in osteosarcoma treatment [32]. *FOXC2-AS1* overexpression in human osteosarcoma tissues correlates with a poor patient outcome and promotes DXR-resistance in vitro using MG63 and KH-OS cell lines [32]. On the other hand, knockdown of *FOXC2-AS1* improves the sensitivity of osteosarcoma cells to DXR. Moreover, *FOXC2* is upregulated in DXR-resistant osteosarcoma cell lines MG63/DXR as well as KH-OS/DXR and human tissues and its levels positively correlate with *FOXC2-AS1*-expression. As with *FOXC2-AS1* knockdown, the inhibition of *FOXC2* results in diminished resistance of osteosarcoma cells deriving from the cell lines MG63/DXR and KH-OS/DXR to DXR. At the same time, FOXC2 contributes to the resistance of osteosarcoma by inducing expression of ABCB1. Therefore, *FOXC2-AS1* and FOXC2 seem to contribute to DXR-resistance in osteosarcoma cells by both enhancing ABCB1-expression [32]. Therefore, *FOXC2-AS1* may not only serve as a predictive biomarker in osteosarcoma indicating resistance to DXR but also as a therapeutic target to increase sensitivity to DXR.

### 2.3. Therapeutic Targets

The lncRNA *MFI2* is overexpressed in osteosarcoma tissue [35]. Its knockdown in osteosarcoma cell-lines MG-63 and Saos-2 reduces proliferation, migration, and invasion of tumor cells, whilst apoptosis is induced. Moreover, *MFI2* seems to regulate Forkhead box P4 (FOXP4), thus promoting proliferation and migration of osteosarcoma cells [35]. Therefore, targeting *MFI2* may be of value in osteosarcoma in order to reduce tumor progression.

The lncRNA *Breast cancer anti-estrogen resistance 4* (*BCAR4*) has both prognostic and therapeutic significance in osteosarcoma [25]. On the one hand, it is overexpressed in osteosarcoma tissues and high levels correlate with advanced stage, large tumor size, presence of lung metastases and poor overall-survival (OS) [25,41]. On the other hand, knockdown of *BCAR4* in-vitro using cell lines MG63 and U2-OS inhibits osteosarcoma cell proliferation and migration by inhibiting the transcription of GLI family zinc finger 2 (GLI2)-dependent target genes. Depletion of *BCAR4* results in reduced transcription of *TGF-beta1*, *IL-6*, *RPS3*, and *MUC5AC*, all being target genes of GLI2 [25]. Consequently, *BCAR4* could serve as a therapeutic target in the treatment of osteosarcoma, as this would inactivate the GLI2 pathway and GLI2-dependent target genes.

Long non-coding RNA *Metastasis-associated lung adenocarcinoma transcript 1* (*MALAT-1*), one of the first lncRNAs identified, is overexpressed in osteosarcoma cell lines Saos-2, U2OS and HOS compared with the osteoblast cell line hFOB1.19 (*p* < 0.05), and is overexpressed in human osteosarcoma tissues [27]. Knockdown of *MALAT-1* results in decreased cell proliferation and migration by inducing cell cycle arrest from the G0/G1 to S-phase and apoptosis in osteosarcoma cell lines U2OS and HOS. Moreover, the knockdown of *MALAT-1* reduces the formation of acting stress fibers and vasculogenic mimicry (VM) in three-dimensional cultures, referring to the ability of osteosarcoma cells to form alternative circulatory systems [27,42]. *MALAT-1* seems to exert its oncogenic function via regulating the Ras homolog gene family, member A (RhoA)/Rho-Kinase (ROCK)-pathway, as downregulation of *MALAT-1* reduces protein levels of RhoA, ROCK1 and 2 in osteosarcoma cell lines U2OS and HOS. Moreover, the downregulation of *MALAT-1* reduces tumorigenesis in mice implanted with osteosarcoma cell lines HOS and U2OS cells transfected with MALAT-1 siRNA in comparison to mice implanted with HOS and USO2 cells transfected with non-specific siRNA. Another effect of *MALAT-1*-knockdown is the inactivation of the Rac1/JNK-pathway via activation of *miR-509* and the downregulation of High-mobility group protein B1 (HMGB1) [43,44]. Both result in reduced osteosarcoma cell growth and induced apoptosis. Taken together, *MALAT-1* may constitute a therapeutic target in the management of osteosarcoma, in order to inhibit cellular proliferation, migration, and invasion.

Another lncRNA involved in cell-cycle regulation is *P21-associated ncRNA DNA damage activated* (*PANDA*), which is overexpressed in the osteosarcoma cell-line U2OS [28]. It is induced by DNA damage following treatment with DXR and etoposide. *PANDA*-depletion reduces the number of viable tumor cells but has no effect on apoptotic cells. Moreover, silencing of *PANDA* leads to an upregulation of cyclin-dependent kinase-inhibitor *p18* mRNA and an accumulation of cells in the G1-phase of the cell-cycle whilst count of cells in the S and G2/M-phase is decreased [28]. Consequently, targeting *PANDA* could be used in practice to induce cell-cycle arrest in the G1/S-phase in osteosarcoma cells.

Other than *MALAT-1* and *PANDA*, lncRNA *Cancer susceptibility candidate 2* (*CASC2*) is significantly downregulated both in human osteosarcoma tissue and osteosarcoma cell lines MG-63, U2OS, SAOS2, and SOSP-9607 (*p* < 0.01; Table 1) [29]. Low *CASC2*-levels correlate with advanced tumor stage. On the other hand, overexpression of *CASC2* is associated with reduced cell proliferation, colony formation and invasion. Of note, *CASC2* usually suppresses *miR-181a* and induces expression of PTEN, ATM, and Ras associated domain family member 6 (RASSF6). The latter one is positively correlated with expression of *CASC2* and consequently expressed at low levels in osteosarcoma. At the same way, experimental overexpression of *CASC2* results in reduced infiltration, colony formation, and cellular growth in osteosarcoma cell-lines [29]. Moreover, in-vivo implantation studies using small interfering-CASC2 resulted in increased tumor weight as compared with implantation of pcDNA-CASC2 [45]. Therefore, *CASC2*-mimics may be used in clinical practice to diminish tumor growth and progression.

*Neuroblastoma associated transcript 1* (*NBAT1*) is likewise downregulated in osteosarcoma tissues and five osteosarcoma cell lines (KHOS, LM7, 143b, USOS, and MG-63) [18]. Low *NBAT1*-levels correlate with clinical stage, presence of distant metastasis and a poor patient outcome. Experimental overexpression of *NBAT1* results in decreased proliferation, migration, and invasion, whilst knockdown leads to enhanced proliferation, invasion, and migration in vitro. In xenograft models, overexpression of *NBAT1* is associated with lower tumor volumes and slow growth, whilst *NBAT1*-silencing results in enhanced tumor growth. Interestingly, *NBAT1* upregulates *PTEN, PDCD4, PTM1*, and *RECK* via an inactivation of *miR-21*, whilst overexpression of *miR-21* reverses this effect [18]. In clinical practice, *NBAT1*-mimics could serve as therapeutic agents to reduce tumor growth and progression.

## 3. Conclusions

Several long non-coding RNAs are involved in the development, progression and invasion of osteosarcoma (Table 1). They may be expressed at lower levels in comparison to normal tissue or osteoblast cell lines, such as *NBAT1, MEG3*, and *CASC2*, or be overexpressed, such as *MFI2, BCAR4*, and *HULC*. They may serve as predictive (e.g., *LUCAT1, FOXC2-*AS1) prognostic (e.g., *FGFR3-AS1, ZEB1-AS1*), therapeutic (e.g., *MALAT-1, PANDA*) and even diagnostic (e.g., *lncRNA-ATB, TUG1*) markers. Especially the latter group is of interest, considering the evolving role of liquid biopsies in the management of osteosarcoma. Liquid biopsies subsume minimally-invasive measures as urine-sampling and blood-tests that are taken to detect and monitor human cancers, with the big advantage for patients of getting by without invasive diagnostic methods as tissue biopsy and explorative surgery. As prognostic biomarkers, lncRNAs can aid prognostication regarding outcome of patients with osteosarcoma, thus eventually guiding treatment decision for or against more aggressive therapeutic approaches. Moreover, predictive lncRNAs may help clinicians to change treatment protocols. Considering that the therapy of patients with osteosarcoma has not significantly changed over the last years, lncRNAs with therapeutic significance may constitute promising targets in the treatment of patients with osteosarcoma. Yet, it has to be borne in mind that far not all lncRNAs can be detected via minimally-invasive methods (e.g., liquid biopsy) and thus still rely on conventional detection methods as tumor biopsy or analysis of the entire tumor specimen. Consequently, further studies are warranted focusing on the applicability of lncRNAs as minimally invasive biomarkers, by detecting them through the blood stream, urine, or even saliva. Moreover, result obtained in experimental studies using osteosarcoma cell lines cannot be simply transferred into the clinical setting. However, these results give a hint towards a potential clinical applicability of lncRNAs, be it as therapeutic targets or prognostic, predictive, and diagnostic biomarkers. 

## Figures and Tables

**Table 1:** Clinical value of lncRNAs involved in the pathogenesis and progression of osteosarcoma

LncRNA (Reference)	Expression	Reported Medium	Test	Clinical Value
MEG3 [22]	Underexpression in comparison to normal adjacent tissue (*p* < 0.05)	Human osteosarcoma tissue samples	qRT-PCR	Prognostic
HULC [23]	Overexpression in comparison to osteoblast cell line hFOB1.19 (*p* < 0.05) and adjacent normal tissue (*p* < 0.05)	Human osteosarcoma tissue; osteosarcoma cell lines MG-63, U2OS, OS-732, and Saos-2	qRT-PCR	Prognostic, therapeutic
LncRNA-ATB [24]	Overexpression in serum samples in comparison to healthy controls (*p* < 0.0001) and in osteosarcoma cell lines vs. hFOB1.19 (*p* < 0.01)	Human osteosarcoma tissue and serum samples; osteosarcoma cell lines U2OS, Saos-2, MG-63, and HOS	qRT-PCR	Diagnostic, prognostic
BCAR4 [25]	Overexpression in comparison to adjacent normal tissue (*p* < 0.001)	Human osteosarcoma tissue samples	qRT-PCR	Therapeutic
FGFR3-AS1 [26]	Overexpression as compared with pair-matched normal tissue (*p* < 0.001)	Human osteosarcoma tissue samples	qRT-PCR	Prognostic
MALAT-1 [27]	Overexpression in comparison to normal tissue and as compared with hFOB1.19 (*p* < 0.05)	Human osteosarcoma tissue samples; osteosarcoma cell lines Saos-2, U2OS, HOS	qRT-PCR	Therapeutic
PANDA [28]	Overexpression as compared with cell lines TIG-3, HeLA, A549, H1299 and MCF7	Osteosarcoma cell line U2OS	qRT-PCR	Therapeutic
CASC2 [29]	Underexpression as compared with non-tumor tissues (*p* < 0.01) and hFOB1.19	Osteosarcoma cell lines MG-63, U2OS, SAOS2, and SOSP-9607	qRT-PCR	Therapeutic
LUCAT1 [30]	Overexpression vs. corresponding parental cell lines (*p* < 0.05)	Methotrexate-resistant osteosarcoma cell line MG63/MTX	qRT-PCR	Predictive
NBAT1 [18]	Underexpression in osteosarcoma tissue samples vs. adjacent normal tissue (*p* < 0.05) and osteosarcoma cell lines in comparison to osteoblast cell line Nhost (*p* < 0.05)	Human osteosarcoma tissue samples; osteosarcoma cell lines KHOS, LM7, 143b, USOS, and MG-63	qRT-PCR	Therapeutic
ZEB1-AS1 [31]	Overexpression in osteosarcoma tissue vs. adjacent normal tissue (*p* < 0.001) and osteosarcoma cell lines vs. hFOB1.19 (*p* < 0.01)	Human osteosarcoma tissue; osteosarcoma cell lines HOS, U2OS, MG63 and Saos-2	qRT-PCR	Prognostic
FOXC2-AS1 [32]	Overexpression in osteosarcoma tissues resistant to doxorubicin vs. non-resistant tissue (*p* < 0.008) and doxorubicin-resistant cell lines vs. doxorubicin-sensitive ones (*p* < 0.05)	Human osteosarcoma tissue samples; doxorubicin-resistant osteosarcoma cell lines MG63/DXR and KH-OS/DXR	qRT-PCR	Predictive
TUG1 [33,34]	Overexpression in osteosarcoma tissue vs. adjacent normal tissue (*p* < 0.01) and osteosarcoma cell lines vs. hFOB1.19 (*p* < 0.01)	Human osteosarcoma tissue and serum samples; osteosarcoma cell lines U2OS, Saos-2, HOS and MG63	qRT-PCR	Diagnostic, prognostic
MFI2 [35]	Overexpression in osteosarcoma tissue samples vs. adjacent normal tissue (*p* < 0.0001)	Human osteosarcoma tissue	qRT-PCR	Therapeutic

lncRNA: Long non-coding RNA; qRT-PCR: quantitative Real-Time PCR.
